# Tunable Acoustic Tweezer System for Precise Three-Dimensional Particle Manipulation

**DOI:** 10.3390/mi15101240

**Published:** 2024-10-08

**Authors:** Jiyun Nan, Hiep Xuan Cao, Jong-Oh Park, Eunpyo Choi, Byungjeon Kang

**Affiliations:** 1 School of Mechanical Engineering, Chonnam National University, Gwangju 61186, Republic of Korea; njy_kiwoon@kimiro.re.kr; 2 Korea Institute of Medical Microrobotics, Gwangju 61000, Republic of Korea; hiep.caoxuan@kimiro.re.kr (H.X.C.); jop@kimiro.re.kr (J.-O.P.); 3 College of AI Convergence, Chonnam National University, Gwangju 61186, Republic of Korea; 4 Graduate School of Data Science, Chonnam National University, Gwangju 61186, Republic of Korea

**Keywords:** acoustic tweezer, acoustic lens, particle manipulation, ultrasonic actuation, targeted drug delivery

## Abstract

This study introduces a tunable acoustic tweezer system designed for precise three-dimensional particle trapping and manipulation. The system utilizes a dual-liquid-layer acoustic lens, which enables the dynamic control of the focal length through the adjustable curvature of a latex membrane. This tunability is essential for generating the acoustic forces necessary for effective manipulation of particles, particularly along the direction of acoustic wave propagation (z-axis). Experiments conducted with spherical particles as small as 1.5 mm in diameter demonstrated the system’s capability for stable trapping and manipulation. Performance was rigorously evaluated through both z-axis and 3D manipulation tests. In the *z*-axis experiments, the system achieved a manipulation range of 33.4–53.4 mm, with a root-mean-square error and standard deviation of 0.044 ± 0.045 mm, which highlights its precision. Further, the 3D manipulation experiments showed that particles could be accurately guided along complex paths, including multilayer rectangular and helical trajectories, with minimal deviation. A visual feedback-based particle navigation system significantly enhanced positional accuracy, reducing errors relative to open-loop control. These results confirm that the tunable acoustic tweezer system is a robust tool for applications requiring precise control of particles with diameter of 1.5 mm in three-dimensional environments. Considering its ability to dynamically adjust the focal point and maintain stable trapping, this system is well suited for tasks demanding high precision, such as targeted particle delivery and other applications involving advanced material manipulation.

## 1. Introduction

Targeted drug delivery (TDD) has emerged as a transformative approach in medical treatment, offering the potential to increase therapeutic efficacy while minimizing adverse side effects [1,2,3]. TDD involves delivering therapeutic agents directly to specific sites within the body, thereby reducing systemic exposure and mitigating associated side effects [4,5,6]. Among the various strategies available for TDD, magnetic drug targeting has been widely explored. This method involves employing magnetic fields to direct drug-loaded magnetic particles to the target site, enabling precise control over drug localization [7,8,9,10,11,12]. However, magnetic drug targeting is constrained by the need to coat drug carriers with magnetic particles, which may affect cell viability and limit broader application of this method [13,14].

In contrast, ultrasound-based methods have gained significant attention due to their noninvasive nature, the possibility of deeper tissue penetration, and the ability to manipulate particles without requiring magnetic coatings [15,16,17,18,19]. Despite these advantages, precise manipulation of particles along the direction of acoustic wave propagation (*z*-axis) remains a considerable challenge. Although traditional acoustic tweezers have been effective in trapping and manipulating particles in planes orthogonal to the direction of wave propagation, they often struggle to achieve precise control along the *z*-axis. Recent advancements in acoustic manipulation techniques have introduced several promising methods, including the use of standing waves [15,16,17], single-beam acoustic fields [20], and phase-adjusted acoustic fields, such as vortex or bottle beams [21,22,23,24,25,26,27,28,29]. These methods, however, often require complex transducer arrays or specially designed acoustic lenses, which can increase system complexity and cost [24,30,31]. In recent years, several studies have focused on particle manipulation using vortex acoustic tweezer or bottle beam acoustic tweezer, but the performance of manipulation along the *z*-axis remains an area of improvement. Ye Yang et al. successfully generated multiple vortex acoustic fields using an arrangement of multiple transducers to manipulate multiple particles [32]. In their study, by adjusting the output phase and amplitude of the transducers, the manipulation range for a single trapped particle reached 10 × 10 × 10 mm^3^. Qinxin Zhou et al. published a study on particle manipulation using a bottle-beam acoustic tweezer [28]. They demonstrated that a single transducer could generate a bottle-beam acoustic field through an acoustic lens and effectively trap particles in a water tank. Additionally, the particle could be manipulated along the *z*-axis by adjusting the acoustic wave frequency, with a manipulable distance of approximately 10 mm in the *z*-axis. In 2024, Teng Li et al. showed that a vortex acoustic tweezer, combined with a robotic arm, could perform four-degree-of-freedom manipulation on a single target, achieving a workspace of 670 × 970 × 250 mm^3^ [33]. In this study, the extended workspace in the *z*-axis was achieved by physically moving the robotic arm.

To overcome these limitations, this study introduces a tunable acoustic tweezer system that incorporates a dual-liquid-layer device. This innovative approach not only allows for dynamic adjustment of the focal length but also enhances acoustic impedance matching between the tweezer system and biological tissues, facilitating more effective particle manipulation. By leveraging the dual-liquid layer as a tunable acoustic lens, our system extends the manipulation range along the *z*-axis, enabling precise control over particle position within a defined three-dimensional space. This research aims to address the shortcomings of existing ultrasound-based manipulation technologies by providing a robust and adaptable solution for three-dimensional particle control. The following sections detail the design, implementation, and experimental validation of the tunable acoustic tweezer system, highlighting its potential applications in TDD and other biomedical fields.

## 2. Acoustic Tweezer System

### 2.1. Acoustic Radiation Force

When an ultrasonic field is applied to a medium containing suspended particles, the particles experience a force known as the acoustic radiation force. This force arises due to the interaction between the acoustic waves and the particles, specifically through the scattering and absorption of the acoustic energy by the particles. The magnitude and direction of the acoustic radiation force depend on several factors, including the properties of the particles (such as size, density, and compressibility) and the characteristics of the acoustic field (such as frequency, amplitude, and wave pattern). For particles that are much smaller than the wavelength of the acoustic waves, the acoustic radiation force can be described in terms of the Gor’kov potential, a theoretical framework that accounts for the monopole and dipole contributions to the force [34]. The Gor’kov potential U is expressed as

(1) $$U=V_{p}\left[\frac{1}{2}\left(k_{0}-k_{p}\right)\langle {\left|p_{1}\right|}^{2}\rangle -\frac{3}{2}\rho_{0}\left(\frac{\rho_{p}-\rho_{0}}{{2\rho }_{p}+\rho_{0}}\right)\langle {\left|v_{1}\right|}^{2}\rangle \right],$$

where $V_{p}$ is the volume of the particle; $k_{p}$ and $k_{0}$ are the compressibility of the particle and the surrounding medium, respectively; $\rho_{p}$ and $\rho_{0}$ are the densities of the particle and the surrounding medium, respectively; $p_{1}$ is the acoustic pressure at the particle’s location; and $v_{1}$ is the velocity of the particle induced by the acoustic field.

The acoustic radiation force F acting on the particle is the negative gradient of the Gor’kov potential:

(2) F=−∇U.

This force tends to move particles toward regions of either high or low acoustic potential, depending on the relative densities and relative compressibility of the particle and the surrounding medium. In most typical scenarios, the particles are drawn to the pressure nodes or antinodes in the standing wave field, where the acoustic radiation force reaches a local extremum. The acoustic radiation force is critical to the operation of the proposed tunable acoustic tweezer system. By carefully designing the acoustic field and tuning the focal length through the dual-liquid-layer device, we can control the distribution and magnitude of the acoustic radiation force; thereby, precise manipulation of particles along the *z*-axis and within a three-dimensional space can be achieved.

### 2.2. Dual-Liquid-Layer Acoustic Lens

**Figure 1:**
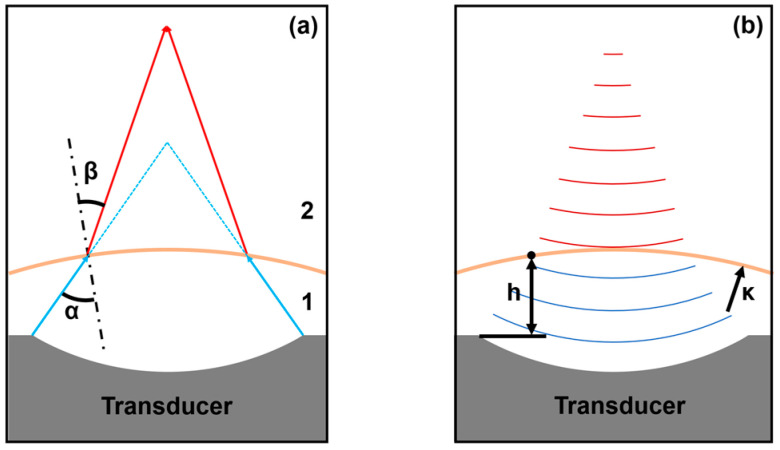
Schematic diagram of the principle of variable focal length of the transducer. (**a**) Schematic of acoustic wave refraction at the interface between two media (medium 1 and medium 2); (**b**) Factors influencing focal length: transducer distance h and membrane curvature κ.

The ability to manipulate particles along the *z*-axis in an acoustic field is significantly enhanced through precise control over the focal length of the acoustic waves. Herein, we introduce a dual-liquid-layer acoustic lens designed to dynamically adjust the focal length of the acoustic tweezer system and thereby extend the range for particle manipulation in three dimensions. The dual-liquid-layer acoustic lens operates on the principle of acoustic wave refraction, which occurs when acoustic waves traverse the interface between two media with different acoustic velocities. According to Snell’s law, the angle of refraction β is determined by the ratio of the acoustic velocities in the two media:

(3) $$\frac{\sin\alpha }{c_{1}}=\frac{\sin\beta }{c_{2}},$$

where α is the angle of incidence, $c_{1}$ is the acoustic velocity in the first medium, and $c_{2}$ is the acoustic velocity in the second medium. By adjusting the curvature of the interface between the two liquids, the focal point of the acoustic waves can be shifted along the *z*-axis; this enables precise control over the position of trapped particles, as shown in Figure 1.

**Figure 2:**
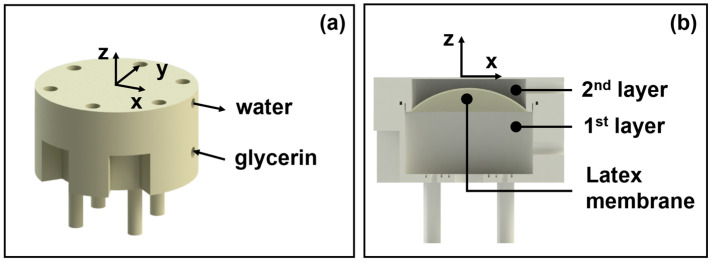
Overview of dual-liquid-layer device. (**a**) Dual-liquid-layer device setup with two immiscible liquids and a latex membrane; (**b**) cross-section showing membrane curvature adjustment by varying liquid volumes.

The dual-liquid-layer device consists of two immiscible liquids, each with distinct acoustic properties, separated by a flexible latex membrane. In our design, glycerin (acoustic velocity ≈ 1920 m/s) and pure water (acoustic velocity ≈ 1480 m/s) are used as the two mediums. The curvature of the latex membrane can be altered by varying the relative volumes of the two liquids, which effectively changes the focal length of the acoustic waves passing through the lens, as depicted in Figure 2. The curvature κ of the latex membrane is a critical factor in determining the focal length of the acoustic lens. As the membrane’s shape approaches that of a spherical crown, the curvature can be described as

(4) R=1/κ,

where R is the radius of curvature of the membrane. The height $h_{m}$ of the spherical crown formed by the membrane is related to the radius of the cross-section $r_{m}$ and the volume of liquid V on one side of the membrane by the following equations:

(5) $$h_{m}=R-\sqrt{R^{2}-{r_{m}}^{2}},$$

(6) $$V=\frac{\pi h_{m}(3r_{m}^{2}+h_{m}^{2})}{6}.$$

Based on these equations, precise control over the membrane curvature can be achieved by adjusting the volume of liquid on either side of the membrane, thereby enabling dynamic tuning of the focal length. The latex membrane is fixed at a set distance from the transducer, and the liquids are contained within a sealed chamber to maintain their respective volumes and the interface curvature. By modulating the liquid volumes via syringes connected to the chamber, the curvature of the membrane—and, consequently, the focal length—can be precisely adjusted. This setup allows the acoustic lens to transition between convex and concave states, providing a versatile means of focusing or defocusing the acoustic waves as needed.

To validate the design and functionality of the dual-liquid-layer acoustic lens, simulations were conducted using COMSOL Multiphysics 5.4 (COMSOL Inc., Los Angeles, CA, USA). These simulations involved modeling the variation in focal length with the membrane curvature under different liquid volume configurations. For the simulations, it was necessary to address the relevance of simulating the influence of the elastic properties of the latex membrane on the acoustic field. Several earlier studies, such as those by Jin-Chen Hsu et al. [35] and Jinhong Guo et al. [36], have investigated the influence of elastic materials on the acoustic field and the method of coupling elastic solid and fluid fields when simulating microfluidic systems based on surface acoustic waves. In this study, however, the primary influence on the acoustic field arises from the refraction caused by the difference in sound speed between the two liquid layers, rather than from the elastic properties of the membrane itself. The elastic membrane used in these experiments is natural latex, with an acoustic impedance between that of water and glycerin. Given its very thin thickness (approximately 0.1–0.2 mm), its effect on the acoustic field at the critical surface between the liquid layers is minimal. Therefore, the influence of the elastic properties of the latex membrane on the acoustic field was not considered in the simulations.

**Figure 3:**
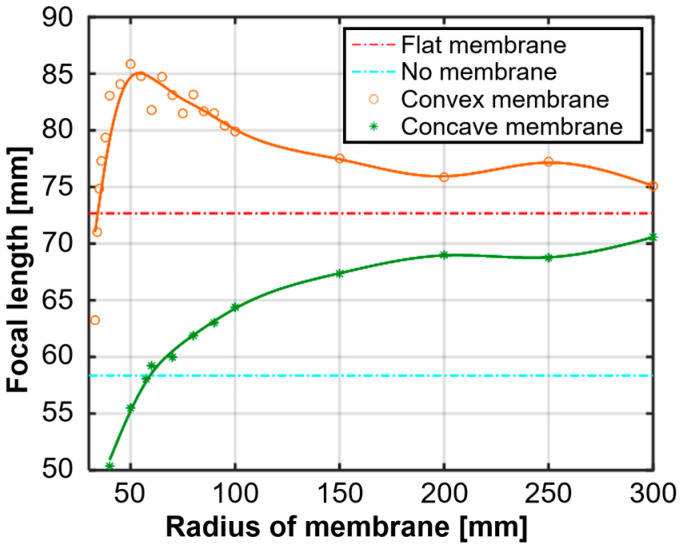
Simulation results of variation in focal length with the membrane’s radius of curvature.

The results of the simulations, illustrated in Figure 3, demonstrate that the focal length can be significantly altered by adjusting the membrane curvature. In the simulations, the focal length of the high-intensity focused ultrasound (HIFU) transducer was approximately 58 mm when the latex membrane was not present. When the membrane was flat, the focal length increased to about 73 mm. Further adjustments to the membrane’s curvature revealed that the focal length could be extended even more by increasing the membrane’s convexity. Specifically, the focal length reached a maximum at a curvature radius of approximately 50 mm, corresponding to a curvature of 0.02 mm^−1^. As the curvature continued to increase, the focal length gradually returned to values closer to those observed with a flat membrane; this demonstrates the possibility of finely tuning the focal length based on the membrane’s curvature. These simulation results confirm the effectiveness of the dual-liquid-layer device in dynamically controlling the focal length of the acoustic tweezer system. This capability is crucial for achieving precise particle manipulation in a three-dimensional space, rendering the system particularly suitable for applications such as TDD.

### 2.3. Fabrication of Acoustic Lens

**Figure 4:**
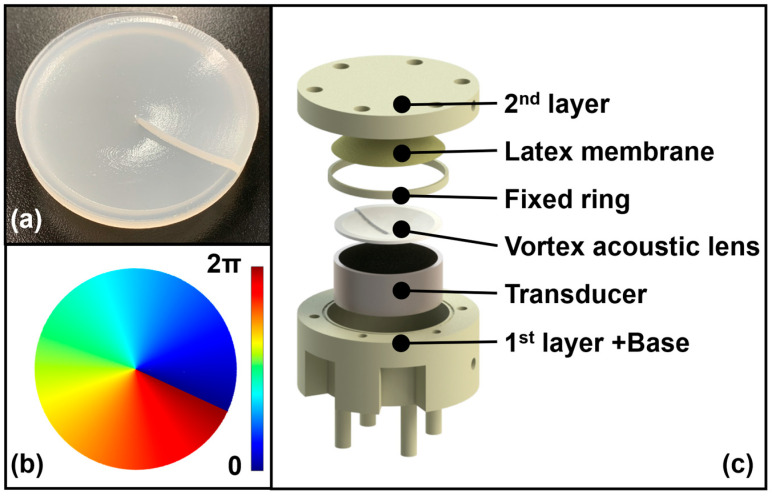
Overview of the tunable acoustic tweezer system. (**a**) Photograph of the fabricated vortex acoustic lens, showing the physical structure and thickness variations; (**b**) Visualization of the phase delay produced by the varying thickness of the vortex acoustic lens; (**c**) Tunable acoustic tweezer system incorporating the fabricated vortex acoustic lens.

The effectiveness of the tunable acoustic tweezer system relies heavily on precise fabrication of the acoustic lens, which is responsible for generating the desired acoustic field for particle manipulation. In this study, a vortex acoustic lens was designed and fabricated to achieve the necessary phase modulation and focal control, as illustrated in Figure 4.

The material chosen for the acoustic lens must possess an appropriate acoustic impedance to ensure efficient transmission of acoustic energy while minimizing reflection losses at the interfaces. The greater the difference in acoustic impedance between materials, the greater the reflection at the interface as the acoustic wave propagates through it. Therefore, in this study, the acoustic impedance of the lens placed between the transducer and the first liquid layer was designed to be as close as possible to the impedance of the transducer’s matching layer (~$1.48\times 10^{6}\;\mathrm{kg}/m^{2}\cdot s$) and the impedance of glycerin (~$2.42\times 10^{6}\;\mathrm{kg}/m^{2}\cdot s$) in the first liquid layer. By doping SiO_2_ powder in silicone rubber, the impedance of the acoustic lens was fine-tuned. The ratio of SiO_2_ powder was adjusted to achieve the required impedance, and the lens was fabricated according to the procedures outlined in [37]. The final impedance of the fabricated lens was $1.54\times 10^{6}\;\mathrm{kg}/m^{2}\cdot s$, which falls within the desired range for minimizing reflection losses. The vortex lens was designed with a specific thickness profile to induce the necessary phase delay across the acoustic wavefront. The thickness difference ∆t required to produce a phase delay of 2π rad (a full wave cycle) was calculated based on the wavelength of the acoustic wave in the lens material and the surrounding medium, as depicted in Figure 4b. The relevant equations for determining the thickness profile are

(7) $$\lambda_{a}=\frac{v_{a}}{f},\;{\;\lambda }_{b}=\frac{v_{b}}{f},$$

(8) $$t_{c}=\frac{\lambda_{a}}{\left|v_{a}-v_{b}\right|},$$

(9) $$\Delta t=t_{c}\lambda_{b}f,\;$$

where $\lambda_{a}$ and $\lambda_{b}$ are the wavelengths of the acoustic waves in the lens material and the medium, respectively; $v_{a}$ and $v_{b}$ are the corresponding acoustic velocities; and f is the frequency of the acoustic waves. The thickness profile of the lens was designed to ensure a gradual phase shift, ultimately creating a vortex acoustic field capable of manipulating particles with high precision. The vortex acoustic lens was fabricated using a precision molding technique, which involved casting the silicone rubber doped with SiO_2_ powder into a mold with the designed thickness profile. The mold was created using a high-resolution 3D printing process to ensure that the intricate geometry of the lens would be accurately reproduced. After being cured, the lens was carefully removed from the mold and subjected to quality checks to verify its dimensional accuracy and surface finish. After being fabricated, the lens was integrated into the tunable acoustic tweezer system, as shown in Figure 4c. The system’s performance was tested by generating the intended vortex acoustic field and assessing its capability to trap and manipulate particles in a three-dimensional space. The successful fabrication and integration of the lens were confirmed by the system’s ability to achieve the desired manipulation targets, thereby validating the design and material selection processes.

## 3. Results and Discussion

### 3.1. Experimental Setup

To validate the performance of the tunable acoustic tweezer system, a comprehensive experimental setup was designed and implemented. This examination involved accurately testing the system’s capability to manipulate particles within a three-dimensional space using the fabricated vortex acoustic lens.

The experimental setup comprised a customized HIFU transducer with diameter of 60 mm, the fabricated vortex acoustic lens, a particle suspension chamber, a two-axis translation stage, and a single-axis translation stage (i-ROBO, Anyang-si, Republic of Korea). The HIFU transducer was driven by a function generator (Agilent 33220A, Santa Clara, CA, USA) and amplified using a high-power amplifier (HSA4101 NF Corporation, Yokohama, Japan) to achieve the desired acoustic power. The particle suspension chamber, filled with degassed water to simulate a biological medium, contained the particles to be manipulated. The entire setup was mounted on a vibration-isolated optical table (DAEIL SYSTEMS, Yongin-si, Republic of Korea) to minimize any external disturbances.

The HIFU transducer was operated at a frequency of 1.15 MHz, chosen to match the resonance conditions required for effective acoustic trapping. The vortex acoustic lens, positioned directly in the matching layer of transducer, modulated the phase of the incoming acoustic waves, generating a vortex field with a well-defined focal point. The particles used in the experiment were acrylic-resin beads with a diameter of 1.5 mm, selected for their high acoustic contrast factor in water. These beads were suspended in the chamber using a gentle stirrer to ensure uniform distribution without introducing significant turbulence. Two charge-coupled device (CCD) high-speed cameras with 2.3 MP color blackfly PoE GigE C-mount (Teledyne FLIR, Wilsonville, OR, USA) were positioned perpendicular to the chamber to visualize and record the particle motion in real time.

The two-axis translation stage was controlled via a precision motorized system, enabling precise positioning of the acoustic tweezer system in the xy-plane relative to the particle suspension chamber. The single-axis translation stage was used to automatically control the volume of glycerin in the dual-liquid-layer device; this allowed for adjusting the focal length of the acoustic lens in real time by changing the curvature of the latex membrane. Data acquisition was handled by a dedicated computer system equipped with LabVIEW (National Instruments, Austin, TX, USA) to control the translation stages, record the video from the high-speed camera, and analyze the movement of the particles.

**Figure 5:**
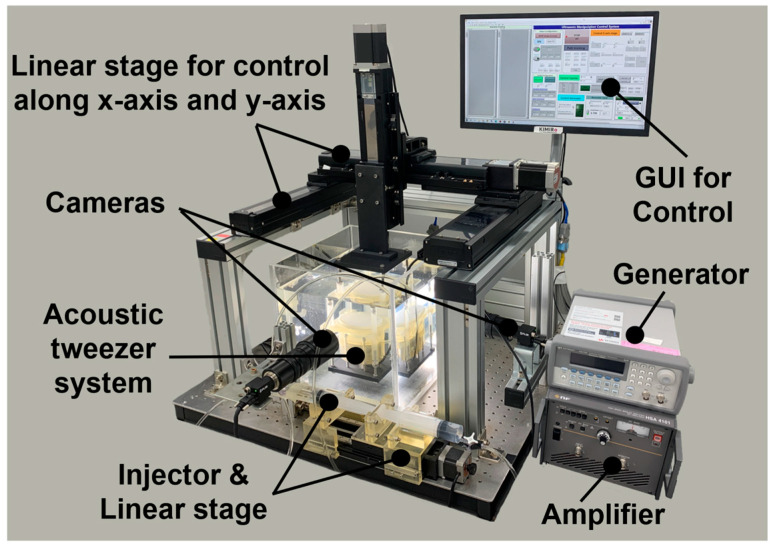
Photograph of experimental setup.

Before the experiments were conducted, the setup underwent a thorough calibration process. The alignment of the HIFU transducer, vortex acoustic lens, and particle suspension chamber was critically adjusted to ensure that the acoustic focal point would align precisely with the center of the chamber. The calibration process involved the use of a laser alignment tool and hydrophone measurements (HNC-0400, Onda, Delaware, OH, USA) to confirm the focal point’s location and the acoustic intensity distribution. An oscilloscope (TELEDYNE LECROY, Chestnut Ridge, NY, USA) was used to monitor the signals and ensure proper synchronization between the components of the equipment. A photograph of the fully calibrated experimental setup is shown in Figure 5. This setup enabled systematic testing across different regions within the three-dimensional space of interest, and the results from these experiments were used to evaluate the system’s performance in trapping and manipulating particles.

**Figure 6:**
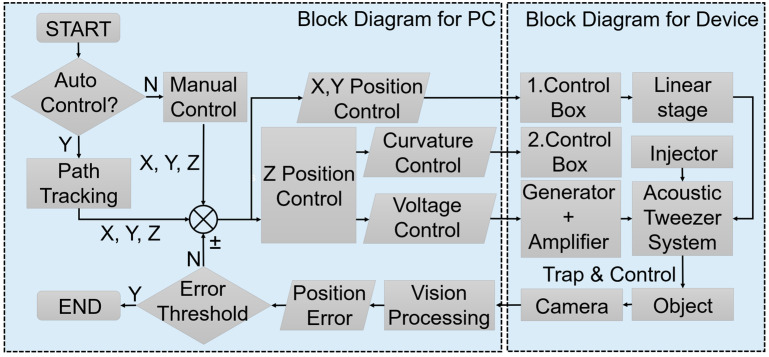
Block diagram of the 3D position control system, illustrating the integration of motorized stages, control software, and feedback mechanisms used to dynamically adjust the focal length and accurately position particles within the experimental setup.

Figure 6 presents a block diagram of the control system used to control and monitor the 3D position of the particles during the experiments. This diagram illustrates the integration of the motorized stages, the control software, and the feedback mechanisms used to dynamically adjust the focal length and position the particles within the desired region of interest.

### 3.2. Scanning Acoustic Field

To characterize and optimize the performance of the tunable acoustic tweezer system, thoroughly understanding the acoustic field generated by the system is essential. The process of scanning the acoustic field involves systematically measuring the acoustic pressure distribution within the region of interest. This measurement helps in visualizing the focal spot, determining the intensity distribution, and identifying any aberrations or asymmetries in the acoustic field.

**Figure 7:**
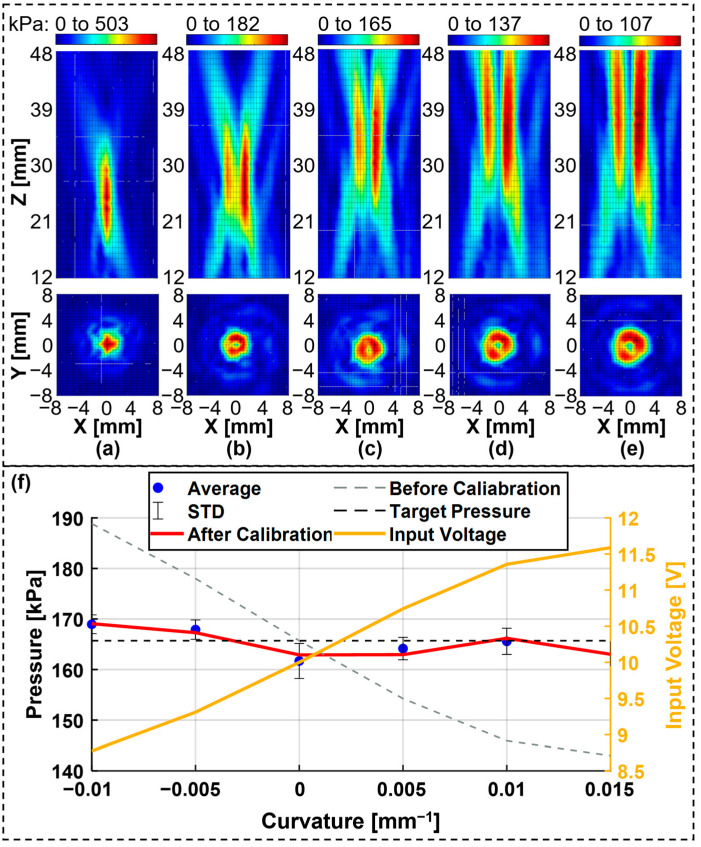
Variation in the focal length of the tunable acoustic tweezer system and calibration result for acoustic pressure at the focal point. (**a**–**e**) Scanning results for the xz and xy planes at the focal point: (**a**) only HIFU was used; (**b**) membrane state was concave with a curvature radius of 100 mm; (**c**) membrane state was flat; (**d**) membrane state was convex with a curvature radius of 100 mm; (**e**) membrane state was convex with a curvature radius of 50 mm; (**f**) acoustic pressure of the vortex acoustic field calibrated by adjusting the voltage input to the HIFU transducer.

The acoustic field was scanned using a calibrated hydrophone mounted on a precision three-axis translation stage. The hydrophone was moved through the region of interest in a grid pattern, with measurements recorded at each grid point to map the acoustic pressure distribution on both the xz and xy planes at the focal point. The scanning process was controlled via the LabVIEW 2017 software program, which ensured accurate positioning of the hydrophone and synchronization with the data acquisition system. During the scanning process, the hydrophone was positioned at various depths along the z-axis to capture the full three-dimensional profile of the acoustic field. The resulting data were processed to generate acoustic field maps, which were then analyzed to assess the focal spot size, the acoustic intensity, and the uniformity of the field. These measurements were crucial for verifying the system’s ability to produce a well-defined focal point with sufficient intensity for particle manipulation. The scanned acoustic field data also provided insights into the effects of adjusting the focal length using the dual-liquid-layer device. By varying the curvature of the latex membrane in the device, different focal lengths were achieved, and the corresponding acoustic field profiles were recorded. Specifically, scanning results were obtained for the following membrane states: only the HIFU without the lens, a concave membrane with a curvature radius of 100 mm, a flat membrane, a convex membrane with a curvature radius of 100 mm, and a convex membrane with a curvature radius of 50 mm. These results are presented in Figure 7a–e. The acoustic pressure was calibrated further by adjusting the voltage input to the HIFU transducer, as shown in Figure 7f.

The results underscore the system’s capability to dynamically adjust the focal point within the desired region, confirming the effectiveness of the tunable acoustic lens design. The data also highlight the variation in acoustic pressure and focal spot characteristics with different membrane curvatures, providing a comprehensive understanding of the system’s performance under various conditions. Overall, the scanning of the acoustic field was a critical step in validating the performance of the tunable acoustic tweezer system. It ensured that the acoustic lens produced the desired focal characteristics and provided the necessary acoustic pressure for effective particle manipulation.

### 3.3. Particle Trapping and Manipulation

**Figure 8:**
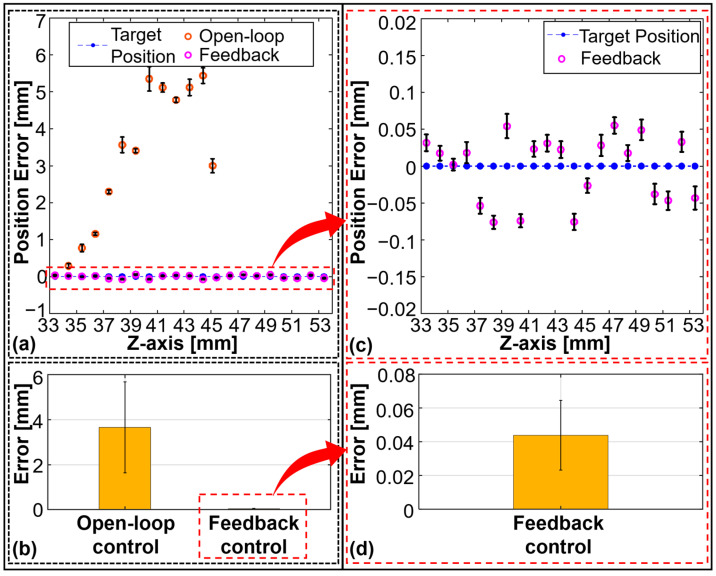
Particle manipulation results and error analysis along the *z*-axis. (**a**) Target positions and real positions of the particle during *z*-axis manipulation using open-loop control and feedback control. (**b**) Error analysis of open-loop control along *z*-axis. (**c**) Target positions and real positions of the particle during *z*-axis manipulation using feedback control. (**d**) Error analysis of feedback control along *z*-axis.

**Figure 9:**
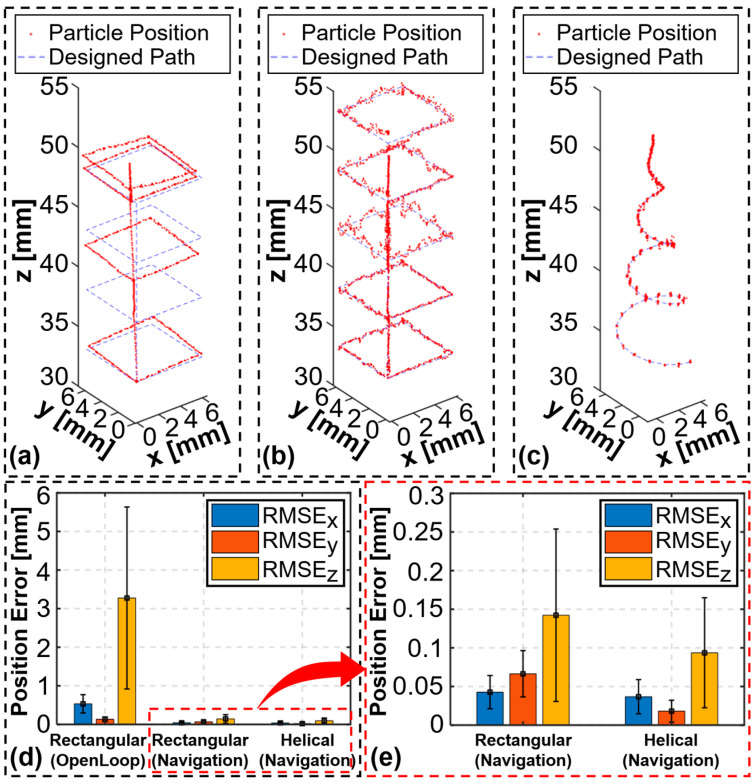
Particle manipulation results and error analysis in 3D: (**a**) open-loop manipulation along a multilayer rectangular path; manipulation using particle navigation system along (**b**) a multilayer rectangular path and (**c**) a helical path; (**d**,**e**) RMSE and STD analysis of the manipulation results for different paths.

In this critical section, we present the experimental results related to the trapping and manipulation of particles using the tunable acoustic tweezer system. The experiments were designed to evaluate the system’s capability to perform both z-axis and 3D particle manipulation with high precision and stability. The findings are supported by detailed quantitative data, as illustrated in Figure 8 and Figure 9.

The ability of the tunable acoustic tweezer system to manipulate particles along the z-axis was first tested under open-loop control conditions. Due to the relatively large dimensions of the acoustic trap, which has a diameter of approximately 1–2 mm (Figure 7b–e), the experiments were conducted using 3D-printed spherical particles with diameters of 1–2 mm. It was determined that particles with a diameter of 1.5 mm could be stably trapped and manipulated. The HIFU transducer was driven by a sinusoidal voltage at 1.15 MHz, with a maximum amplitude of 60 Vpp. The coordinate system used was consistent with that depicted in Figure 2, with the origin at the center of the upper surface of the dual-liquid-layer device. To achieve stable z-axis manipulation, the relationship between the membrane curvature of the dual-liquid-layer device and the focal length was established through repeated scans of the acoustic pressure field. This enabled the focal length to be dynamically adjusted based on a target input. The results of z-axis manipulation using open-loop control are shown in Figure 8a,b. Figure 8a displays the target positions (blue dotted line) and the mean (red circles) and standard deviation (STD) of the real-time position of the particle recorded when repeating the experiment 10 times along the z-axis, with a spacing of 1 mm between adjacent target positions. The manipulation range achieved was 33.4–45.4 mm. The error analysis in Figure 8b shows the accuracy of the open-loop control system, which exhibited a root-mean-square error (RMSE) and STD of 3.660 ± 2.021 mm. A recording of the experiment is available in Video S1.

To enhance the precision of particle manipulation, a visual feedback-based particle navigation system was developed. This system improves z-axis control by adjusting the input voltage to the HIFU transducer, with focal length adjustments extending the range of stable manipulation. The particle navigation system automatically adjusts the voltage to bring the particle within a set error threshold of ±0.1 mm. The results, presented in Figure 8c,d, demonstrate a significant improvement in accuracy. The manipulation range was extended to 33.4–53.4 mm, with a much lower RMSE and STD of 0.044 ± 0.045 mm. A recording of the experiment is available in Video S2.

The system’s 3D manipulation capabilities were further evaluated by combining z-axis control with movements along the x- and y-axes using a linear stage. The particle navigation system was employed to manipulate particles along predefined paths, including a multilayer rectangular path and a helical path. The results of these experiments are presented in Figure 9.

In the open-loop manipulation along the multilayer rectangular path (Figure 9a,d), the particle was moved within a 6 × 6 × 15 mm^3^ volume, with RMSE and STD values of 0.533 ± 0.235 mm, 0.132 ± 0.060 mm, and 3.276 ± 2.359 mm along the x-, y-, and z-axes, respectively. These results show significant offsets, particularly along the *z*-axis, due to voltage control limitations. When the particle navigation system was used (Figure 9b,e), the manipulation along the same multilayer rectangular path was significantly improved, with the particle confined to a 6 × 6 × 20 mm^3^ volume and the RMSE and STD reduced to 0.043 ± 0.022 mm, 0.066 ± 0.030 mm, and 0.142 ± 0.112 mm along the x-, y-, and z-axes, respectively. Additionally, the particle was successfully manipulated along a helical path (Figure 9c,e) within a 4 × 4 × 18 mm^3^ volume, with RMSE and STD values of 0.037 ± 0.022 mm, 0.018 ± 0.014 mm, and 0.094 ± 0.071 mm along the x-, y-, and z-axes, respectively. These results indicate that while open-loop manipulation was prone to significant offsets, particularly along the *z*-axis, the particle navigation system effectively mitigated these issues, ensuring high precision even on complex 3D trajectories. Recordings of the experiment are available in Videos S3–S5.

## 4. Conclusions

In this study, we successfully developed and validated a tunable acoustic tweezer system capable of precise particle trapping and manipulation in a three-dimensional space. The system’s design leverages a dual-liquid-layer acoustic lens, allowing for dynamic control of the focal length by adjusting the curvature of a latex membrane. This tunability is key to achieving the necessary acoustic forces for effective particle manipulation, particularly along the *z*-axis.

Our experiments demonstrated that the system could stably trap and manipulate spherical particles with diameters as small as 1.5 mm. The system’s performance was thoroughly evaluated through a series of *z*-axis and 3D manipulation experiments. In the *z*-axis experiments, a manipulation range of 33.4–53.4 mm was achieved when using the visual feedback-based particle navigation system, with an impressive RMSE and STD of 0.044 ± 0.045 mm. This level of precision underscores the proposed system’s capability to control particle position with high accuracy. The 3D manipulation experiments further validated the system’s robustness. Particles were successfully guided along complex paths, including multilayer rectangular and helical trajectories, with minimal deviation. The particle navigation system proved particularly effective in reducing the errors associated with the open-loop control, significantly enhancing the accuracy of particle positioning along all three axes. The best performance was observed during the helical path manipulation, where the RMSE and STD were minimized to 0.037 ± 0.022 mm, 0.018 ± 0.014 mm, and 0.094 ± 0.071 mm along the *x*-, *y*-, and *z*-axes, respectively.

In conclusion, the feasibility demonstrated in this study shows that the tunable acoustic tweezer system is a powerful tool for precise and flexible particle manipulation. Its ability to dynamically adjust the focal point in real time, combined with the effectiveness of the visual feedback-based particle navigation system, makes it well suited for applications requiring fine control over particles in three-dimensional environments. This research represents a significant advancement in the field of acoustic manipulation, offering a versatile and accurate platform for applications that demand precise spatial control over particle positioning, such as targeted delivery systems or microstructure assembly. While the proposed mechanism can achieve precise particle trapping and manipulation in a three-dimensional space, this study has some limitations. The experimental procedures validated in this study were conducted in water, without considering the flow dynamics and higher viscosity present in real-world biological media. Future research will focus on improving the system’s stability at higher *z*-axis positions and enhancing the anti-disturbance capabilities of the navigation system, particularly under conditions where external influences (such as fluid flow) may affect particle stability. Furthermore, additional investigations will be carried out to explore the potential of the acoustic tweezer system in developing novel targeted drug delivery applications in biological environments.

## Data Availability

The original contributions presented in the study are included in the article/Supplementary Materials, further inquiries can be directed to the corresponding authors.

## Supplementary Materials

The following supporting information can be downloaded at: https://www.mdpi.com/article/10.3390/mi15101240/s1, Video S1: Trapping and open-loop manipulation of a particle along the *z*-axis; Video S2: Trapping and manipulation of a particle along the *z*-axis using the particle navigation system; Video S3: Open-loop manipulation of a particle along a multilayer rectangular path; Video S4: Manipulation of a particle along a multilayer rectangular path using the particle navigation system; Video S5: Manipulation of a particle along a helical path using the particle navigation system.
